# Acute toxicity assessment and metabolomic profiling of *Taxus sumatrana* leaf extract as a sustainable alternative to bark harvesting

**DOI:** 10.1016/j.toxrep.2026.102289

**Published:** 2026-06-06

**Authors:** Pamungkas Rizki Ferdian, Wanda Kuswanda, Hadi Sukardi Alikodra, Herjuno Ari Nugroho, Haidir, Ni Luh Putu Rischa Phadmacanty, Ardya Widyastuti, Rizki Rabeca Elfirta, Rini Madyastuti Purwono, Asep Hidayat, Yani Silfariani, Alif Zikri Fadhali

**Affiliations:** a Research Center for Applied Zoology, National Research and Innovation Agency Republic of Indonesia, Bogor, West Java, Indonesia; b Research Center for Biota Systems, National Research and Innovation Agency Republic of Indonesia, Bogor, West Java, Indonesia; c Bogor Natur Indonesia, Bogor, West Java, Indonesia; d Research Center for Applied Microbiology, National Research and Innovation Agency Republic of Indonesia, Bogor, West Java, Indonesia; e Kerinci Seblat National Park Agency, Ministry of Forestry of Indonesia, Sungat Penuh, Jambi, Indonesia; f Dialovet Animal Care, Bogor, West Java, Indonesia; g School of Veterinary and Biomedicine, IPB University, Bogor, West Java, Indonesia; h Research Center for Ecology, National Research and Innovation Agency Republic of Indonesia, Bogor, West Java, Indonesia

**Keywords:** Acute toxicity, Bioprospecting, LD_50_, Metabolomic profiling, *Taxus sumatrana*

## Abstract

*Taxus sumatrana* (Taxaceae) is an Indonesian endemic species known for its anticancer activity due to taxane derivatives. Despite its pharmacological importance, *T. sumatrana* remains underexplored, and its populations are increasingly threatened by extensive bark harvesting, compromising the tree’s natural defence system and often leads to stem rot and mortality. This risk is increasing since the species has slow generative and vegetative growth. The use of regenerable plant parts, such as leaves, represents a more sustainable alternative. This study aimed to evaluate the *in vivo* acute oral toxicity of ethanolic *T. sumatrana* leaf extract in mice to support its safe and sustainable utilization. Metabolomic profiling was also conducted using UPLC-HRMS/MS, followed by *in silico* toxicity prediction. *In vivo* evaluation included mortality observation, relative organ-to-body weight ratio analysis, blood chemistry assessment, and histopathological examination. No mortality was observed within 24 h of administration, indicating an LD₅₀ greater than 2000 mg/kgBW. *In silico* toxicity prediction showed that all 100 identified metabolites were predominantly non-AMES toxic, non-inhibitors of hERG I/II channels, and had predicted acute LD₅₀ values exceeding 2000 mg/kgBW. Relative organ weights and blood chemistry parameters exhibited only minor variations across treatment groups, reflecting a favourable physiological response. Histopathological evaluation revealed liver and kidney abnormalities below 20%, indicating minimal and tolerable tissue alterations. These findings support the use of leaves as a safe and sustainable alternative to bark harvesting. Further toxicity studies particularly sub-chronic and chronic toxicity evaluations are recommended to reach a comprehensive conclusion on the safe use of the leaf extract.

## Highlights

• Acute oral toxicity of *Taxus sumatrana* leaf extract was evaluated in mice.
• No mortality observed up to 2000 mg/kgBW (LD50 > 2000 mg/kgBW).
• Organ weights, blood chemistry, and histopathology showed minimal alterations.
• UPLC-HRMS/MS identified 100 compounds in the leaf extract.
• Leaves offer a safer and sustainable alternative to bark harvesting.

## Graphical Abstract

**Figure:**
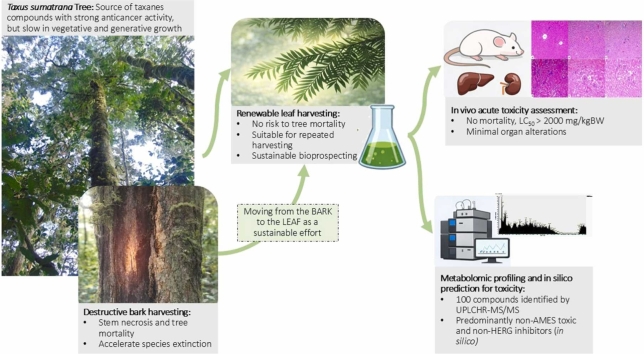

## 1 Introduction

*Taxus sumatrana*, a member of the Taxaceae family, is widely recognized for its medicinal value, particularly its anticancer potential. Its pharmacological activity is primarily attributed to taxane derivatives, including paclitaxel (Taxol®), docetaxel (Taxotere®), and cabazitaxel (Jevtana®), which exhibit pronounced cytotoxic and therapeutic effects against various cancer types [1], [2], [3]. These bioactive compounds have been detected in several plant organs, including the bark, young stems, and leaves of Taxaceae species [4], [5]. Despite this broad distribution, the bark has historically been the most intensively exploited plant part due to its high taxane content [6], [7]. However, bark harvesting poses a serious threat to species survival, as the bark functions as the tree’s primary protective barrier. Its removal exposes the stem to parasitic organisms and microbial pathogens, accelerates tissue damage, and ultimately increases the risk of tree mortality.

In recent years, the utilization of *Taxus* has increased substantially in areas surrounding Kerinci Seblat National Park (KSNP), where local communities increasingly rely on the species after recognizing its medicinal benefits [8]. This growing demand has contributed to a marked decline in natural populations of *T. sumatrana*, driven largely by over-exploitation through bark harvesting [7,8]. In response, *T. sumatrana* has been listed in Appendix II of the Convention on International Trade in Endangered Species of Wild Fauna and Flora (CITES) and is protected under Indonesian national legislation, specifically Regulation of the Minister of Environment and Forestry of the Republic of Indonesia No. P.106/MENLHK/2018 (the second amendment to Regulation No. P.20/MENLHK/2018 concerning protected plant and animal species) [33]. Nevertheless, legal protection alone has not been sufficient to prevent population decline, as illegal harvesting persists due to the high economic value of the bark and the limited availability of the species in natural habitats.

The vulnerability of *T. sumatrana* is further exacerbated by its inherently slow growth rate during both generative and vegetative phases, which restricts natural regeneration and increases the risk of local extinction. Current population estimates within KSNP indicate densities of only 21–50 individuals per hectare, with clustered distributions confined to specific locations such as Mount Kerinci, Mount Tujuh, and the Alahan Panjang Forest [9]. To ensure long-term species survival, integrated conservation strategies are required, combining in situ protection with ex situ cultivation. The development of ex situ plantations on community-managed lands has been proposed as an effective approach to support population recovery while simultaneously reducing harvesting pressure on wild populations [8].

Beyond conservation, ex situ cultivation also enables sustainable utilization by providing renewable sources of bioactive compounds for medicinal development. Since the discovery of taxane compounds in the 1990s, research on *Taxus* species has expanded from compound identification to cultivation and production strategies [10]. However, studies on *T. sumatrana* have predominantly focused on anticancer activity derived from bark tissues [1]. More recent investigations have reported the presence of additional bioactive constituents in both the bark and leaves of *T. sumatrana*, including phenolic compounds, lignans, and flavonoids [11], suggesting that non-bark tissues, particularly leaves, may serve as viable alternative sources of pharmacologically active compounds. In term of biological activities, while bark extracts are conventionally recognized for their anticancer properties, recent pharmacological comparisons indicate that leaf extracts also exhibit significant and comparable cytotoxicity. For instance, studies have demonstrated that extracts from both the bark and leaves of *T. sumatrana* are highly active against various human cancer cell lines, including HeLa, T47D, and MCF−7 [12]. Furthermore, the abundance of phenolic and flavonoid compounds in the leaves confers additional antioxidant and antimicrobial benefits that are less prominent in the bark [10], [11]. This highlights that leaves are not merely a substitute source of taxanes, but a novel, multifaceted pharmacological resource in their own right.

Historically, bark has been the primary source of taxol because it provides the highest average yield among plant parts [13]. For instance, in *T. brevifolia*, bark exhibits an average taxol yield of 0.015% (ranging from 0.0001% to 0.069%), which is notably higher than the 0.0015% average yield found in its leaves [13]. Furthermore, *T. brevifolia* bark generally yields approximately 100 mg of taxol per kg of bark [14]. However, despite the generally lower average yield in foliage, surveys of leaves from various *Taxus* species, including *T. sumatrana*, have demonstrated that taxol yields can actually be comparable to those of dried *T. brevifolia* bark [12], [14]. Additionally, the leaves serve as a renewable source for other valuable taxol precursors, such as 10-deacetylbaccatin III [14]. Because leaves can be harvested continuously without causing irreversible damage to the tree, this long-term pharmacological and ecological viability firmly justifies the shift toward utilizing leaves as a sustainable alternative to destructive bark harvesting.

Despite this advantage, scientific evidence supporting the safety of *T. sumatrana* leaf extracts remains limited, particularly in terms of toxicological evaluation. Toxicity assessment is essential to ensure safe use in animals and humans, provide guidance for dosage determination, and identify potential target organ effects. Acute toxicity studies offer fundamental baseline information by evaluating immediate physiological, biochemical, and histopathological responses following exposure [15]. These in vivo assessments are further strengthened when combined with in silico toxicity predictions, which help anticipate potential toxicological risks associated with identified metabolites [16], [17].

Accordingly, the present study aims to assess and evaluate the acute toxicity of ethanolic leaf extracts of *T. sumatrana* using an integrated *in vivo* and *in silico* approach. The observation period was limited to 24 h rather than the conventional 7–14 days to specifically capture immediate toxicological responses while minimizing confounding recovery-related effects. By focusing on leaf extracts, this study represents an initial step toward shifting utilization from bark to a sustainable, renewable plant part. The findings are expected to provide essential safety data that support conservation-oriented utilization and inform future pharmaceutical and biomedical applications of *T. sumatrana*.

## 2 Material and method

### 2.1 Ethical permissions

Permission for the collection of *T. sumatrana* leaves, a protected plant species, was granted by the Directorate of Natural Resources and Ecosystems, Ministry of Environment and Forestry of the Republic of Indonesia, under document number SK.144/KSDAE/SETKSDAE/KSA.2/7/202. The plant material was collected from Kerinci Seblat National Park, Jambi, Indonesia, in accordance with national conservation regulations. All experimental procedures involving animals were approved by the Ethical Clearance Committee for Animal Experimentation of the National Research and Innovation Agency (BRIN), Indonesia, under approval number 097/KE.02/SK/05/2025. The approved procedures covered all aspects of the study, including animal housing and husbandry, feeding, dose administration, data collection, euthanasia, and organ sampling.

### 2.2 Extract preparation

The leaves of *T. sumatrana* were collected from several traditional nurseries (approximately 1500m-2000m a.s.l.) around Kerinci Seblat National Park, Jambi, Indonesia. Samples were then transferred to the Laboratory of National Research and Innovation Agency of Indonesia (BRIN) Cibinong, West Java, Indonesia for further processing. The samples were cleaned and dried at 50°C for 7 days. Dried samples were ground and sieved using a 60-mesh sieve to obtain fine powder. The dried leaf powder of *T. sumatrana* was extracted using 70% ethanol at a 1:100 (w/v) rasio for 3 days on a labotatory shaker. The extract solution was filtered using filter paper. The filtrate was concentrated using a rotary evaporator at 40°C until dry and solvent-free. This process resulted in a final extract yield of 27.60 ± 4.67%. The final extract was stored at 4°C until further use.

### 2.3 Metabolomic profiling

Metabolomic profiling was performed using ultra-high-performance liquid chromatography coupled with high-resolution tandem mass spectrometry (UPLC–HRMS/MS). Prior to analysis, the solvent-free extract was dissolved in methanol to ensure chromatographic compatibility and subsequently filtered through a 0.22 μm PTFE membrane filter. Chromatographic separation was carried out using a binary mobile phase system consisting of MS-grade water with 0.1% formic acid (A) and MS-grade methanol with 0.1% formic acid (B), applied in a gradient elution mode at a flow rate of 0.3 mL/min. The gradient program began with 5% B, which was linearly increased to 90% B over 16 min, maintained at this composition for 4 min, and then returned to the initial condition (5% B) for re-equilibration over 25 min. The column temperature was maintained at 40 °C, and the injection volume was set at 3 μL.

Mass spectrometric analysis was conducted using an untargeted acquisition strategy with full MS and data-dependent MS/MS (dd-MS²) modes, operated in both positive and negative ionization polarities. Nitrogen was employed as the sheath gas, auxiliary gas, and sweep gas, with respective flow settings of 32, 8, and 4 arbitrary units (AU). The spray voltage was set to 3.30 kV, the capillary temperature to 320°C, and the auxiliary gas heater temperature to 300°C. Mass spectra were acquired over an *m/z* range of 66.7–1000, with a resolution of 70,000 for full MS and 17,500 for dd-MS². Instrument control and data acquisition were performed using Xcalibur™ software version 4.4 (Thermo Fisher Scientific, Bremen, Germany).

The UPLC-HRMS/MS analysis was performed in technical duplicates to ensure data consistency and reproducibility. Post-acquisition data processing, including compound identification and annotation, was conducted using Compound Discoverer software version 3.2 (Thermo Fischer Scientific). The acquired MS/MS spectra were queried against comprehensive spectral databases, specifically mzCloud and ChemSpider. Putative compound annotation was accepted based on strict identification criteria, applying a precursor mass error tolerance of within ± 3.0 ppm to ensure high-confidence metabolic profiling.

### 2.4 Prediction for toxicity

The toxicity of compounds identified by UPLC–HRMS/MS was evaluated using an in silico prediction approach implemented through the pkCSM online platform (https://biosig.lab.uq.edu.au/pkcsm/) [18]. Prior to analysis, the chemical structures of the identified compounds were drawn using ChemSketch software and subsequently converted into Simplified Molecular Input Line Entry System (SMILES) format, as required by the prediction tool. Toxicity prediction focused on parameters relevant to acute toxicity assessment. These parameters included AMES toxicity, human ether-a-go-go–related gene (hERG) inhibition, acute lethal dose (LD₅₀), hepatotoxicity, and maximum tolerated human dose. The in silico evaluation was performed to provide a preliminary assessment of the potential toxicological risks associated with the identified metabolites.

### 2.5 Animal model

Three-week-old female DDY mice (n = 20) with 25–30 g body weight were used as experimental animals in this study. The animals were obtained from a licensed local supplier and housed at the Animal Facility of the Teaching Animal Hospital, IPB University, Indonesia. Throughout the experiment, mice were maintained under controlled environmental conditions, with free access to standard laboratory feed and water ad libitum, a 12-hour light and 12-hour dark cycle, humidity 75 ± 5%, and temperature 25 ± 2 °C. Upon arrival, all animals underwent a 7-day acclimatization period under veterinary supervision to ensure physiological stability prior to experimentation. During the acclimatization period, mice received antiparasitic treatment as part of routine health management.

### 2.6 Acute oral toxicity using fixed dose method based on OECD 420 guideline

The in vivo acute toxicity study was carried out in accordance with OECD Guideline 420 (Fixed Dose Method) and the Regulation of the Indonesian Food and Drug Authority (BPOM) No. 10 of 2022, with minor modifications [15], [19], [20] The experimental design followed two sequential stages, consisting of a sighting study and a main study, to determine appropriate dose levels and evaluate potential acute toxic effects. In the sighting study, one animal was used for each dose level, which included 0 mg/kg body weight (BW) as placebo, 5 mg/kg BW, 50 mg/kg BW, 300 mg/kg BW, and 2000 mg/kg BW. Each dose was administered on a separate day to allow a full 24-hour observation period, as required for acute toxicity assessment. This preliminary phase was intended to ensure animal safety and to guide dose selection for the subsequent main study.

Based on the absence of mortality or observable toxicity signs in the sighting study, the main study was conducted using 15 rats, which were randomly allocated into three groups with five animals per group. The selected treatment groups received placebo, 300 mg/kgBW, or 2000 mg/kgBW, in line with OECD 420 recommendations and the lack of prior toxicity data on *T. sumatrana* leaf extract. Each animal received a single oral administration, while the placebo group was administered sterile distilled water (aquadest). Following dosing, animals were carefully monitored at 1, 4, 8, 12, and 24 h post-treatment. Clinical observations focused on changes in general appearance and behavior, including fur condition, body weight loss, anorexia, lethargy, tremors, seizures, diarrhea, and vomiting.

After completion of the 24-hour observation period, all animals were euthanized using an overdose of pentobarbital (Valabarb®; 150 mg/kg BW) administered via intraperitoneal injection [15], [21]. Blood samples were collected through cardiac puncture for further analysis. Subsequently, the right kidney and liver were carefully excised and weighed. The collected organs were fixed in 10% neutral buffered formalin (NBF) to preserve tissue integrity for histopathological examination. In addition, the organ-to-body-weight ratios of the liver and right kidney were calculated as supporting indicators of acute toxicological effects.

### 2.7 Blood chemistry analysis

Blood samples were collected by cardiac puncture from euthanized mice immediately after the acute toxicity assessment. The collected blood was allowed to clot and subsequently centrifuged at 4000 rpm to obtain serum for biochemical analysis. Serum samples were analyzed using a Catalyst One® Chemistry Analyzer (IDEXX Laboratories, Inc., USA) in accordance with the manufacturer’s instructions. The evaluated biochemical parameters were selected based on their relevance to hepatic and renal toxicity. These parameters included total protein, albumin, globulin, blood urea nitrogen (BUN), creatinine, alanine aminotransferase (ALT), and alkaline phosphatase (ALKP).

### 2.8 Histopathological analysis

Histopathological specimens were prepared from the preserved organ tissues. The samples were processed for histological examination using the paraffin-embedding method, beginning with washing under running tap water, followed by dehydration through a graded series of ethanol solutions. The tissues were subsequently embedded in paraffin and sectioned at a thickness of 5 μm using a rotary microtome (Yamato Scientific Co., Ltd., Tokyo, Japan). The resulting sections were deparaffinized using graded xylene washes, rehydrated, and then stained with hematoxylin and eosin (H&E). All stained sections were cleared in xylene and mounted with Entellan® (Merck Millipore).

Histopathological evaluation was performed using a compound light microscope (Olympus Corporation, Tokyo, Japan) equipped with a digital camera connected to a personal computer. Representative micrographs were captured at 10x and 20x magnifications for documentation and analysis. The assessment focused on structural and cellular alterations indicative of tissue injury or toxicological effects. To eliminate observer bias, the evaluation was conducted in a strict blinded manner by a veterinary pathologist who unaware of the specific treatment groups. The severity of tissue lesion (including cellular necrosis, steatosis, and inflammatory infiltration) was quantified using a standardized semi quantitative scoring system on a 0–4 scale. The score were defined base on the percentage of field involvement across multiple randomized microscopic fields per tissue section; Score 0 = normal (no observed lesions); Score 1 = minimal/mild (< 25% field involvement); Score 2 = moderate (26–50% field involvement); Score 3 = marked (51 – 75% field involvement); Score 4 = severe ( > 75% field involvement). The histological findings were used to support the evaluation of organ specific toxicity.

### 2.9 Statistical analysis

Data from organ-to-body weight ratios and blood chemistry parameters were analyzed using appropriate statistical methods. Data sets that did not meet the assumptions of homogeneity of variance were analyzed using non-parametric approaches. The Kruskal–Wallis test was applied, followed by Dunn’s post hoc test when significant differences were observed. This approach was used for albumin and alkaline phosphatase (ALKP) parameters. All remaining data that satisfied the assumptions for parametric analysis were evaluated using one-way analysis of variance (ANOVA), with Tukey’s post hoc test applied for multiple comparisons. A significance level of 95% was used for all statistical analyses. Statistical analyses were performed using RStudio (version 2025.9.01). Data visualization and statistical processing were supported by the ggplot2, rstatix, and FSA packages.

## 3 Results

### 3.1 Metabolomic profiling

Untargeted metabolomic analysis of the ethanolic leaf extract of *Taxus sumatrana* led to the annotation of 100 compounds belonging to diverse chemical classes, including amino acids, organic acids, alkaloids, phenolic compounds, flavonoids, terpenoids, fatty acids, and lipid derivatives (Table 1). The detected metabolites were distributed across a wide chromatographic range, with retention times spanning from 0.68 to 15.59 min and molecular weights ranging between 103.10 and 592.27 g/mol. Among all identified compounds, 2-methoxyresorcinol was the most abundant metabolite, accounting for 25.12% of the total relative abundance. Other metabolites present at comparatively high levels included choline (9.31%) and coumarin (2H-chromen−2-one) (8.33%). Together, these findings indicate that the leaf extract contains a complex mixture of polar and semi-polar metabolites with varied abundance profiles. It is important to note that untargeted UPLC-MS/MS analysis provides putative compound annotations based on spectral library matching. Consequently, compounds containing nitrogen (N) and phosphorous (P) detected in the early retention times (i.e. choline and glycerophosphoglycerol) are ubiquitous primary metabolites essential for plant cell structure, particularly as precursors for phosphatidylcholine and chloroplast thylakoid lipids. Conversely, certain complex annotations, such as specific sulfur-containing derivatives, represent the highest-scoring spectral metches and may encompass closely related structural isomers.

**Table 1:** Untargeted metabolomic profile by UPLCHR-MS/MS.

No	Compound	Molecular Formula	Molecular Weight (g/mol)	Retention Time (minute)	Relative Abundance (%)
1	.alpha.-Aminoadipic acid	C_6_H_11_N O_4_	161.07	0.68	0.35
2	Choline	C_5_H_13_NO	103.10	0.76; 0.86	9.31
3	4-guanidinobutanal	C_5_H_11_N_3_O	129.09	0.77	0.03
4	Dihydrothymine	C_5_H_8_N_2_O_2_	128.06	0.78	0.67
5	D-(+)-Pipecolinic acid	C_6_H_11_NO_2_	129.08	0.78	0.39
6	N-Acetyl-S-(allylcarbamothioyl)cysteine	C_9_H_14_N_2_O_3_S_2_	262.05	0.78	0.06
7	DL-Stachydrine	C_7_H_13_NO_2_	143.09	0.78	0.10
8	Betaine	C_5_H_11_NO_2_	117.08	0.78; 0.88	0.80
9	Glycerophosphoglycerol	C_6_H_15_O_8_P	246.05	0.79	0.09
10	1-Aminocyclohexanecarboxylic acid	C_7_H_13_NO_2_	143.09	0.79; 0.89	0.24
11	DL-Carnitine	C_7_H_15_NO_3_	161.11	0.85	0.15
12	4-Guanidinobutyric acid	C_5_H_11_N_3_O_2_	145.09	0.87	0.63
13	Valine	C_5_H_11_NO_2_	117.08	0.88	0.43
14	D-(+)-Proline	C_5_H_9_NO_2_	115.06	0.88	0.39
15	Benzoquinone	C_6_H_4_O_2_	108.02	0.90	0.44
16	5-Hydroxymethyl−2-furaldehyde	C_6_H_6_O_3_	126.03	0.93	0.88
17	Nicotinic acid	C_6_H_5_NO_2_	123.03	0.97	0.37
18	Nicotinamide	C_6_H_6_N_2_O	122.05	0.99	0.43
19	L-Pyroglutamic acid	C_5_H_7_NO_3_	129.04	1.03	1.20
20	Glycolophenone	C_8_H_8_O_2_	136.05	1.10	0.12
21	L-Tyrosine methyl ester	C_10_H_13_NO_3_	195.09	1.20	0.74
22	2H-chromen−2-one	C_9_H_6_O_2_	146.04	1.32; 2.75; 3.00; 4.10; 4.20; 4.78	8.33
23	m-Anisaldehyde	C_8_H_8_O_2_	136.05	1.43	0.33
24	(R)-Salsolinol	C_10_H_13_NO_2_	179.09	1.59	0.36
25	3,4-Methylenedioxyamphetamine	C_10_H_13_NO_2_	179.09	1.59	0.40
26	2-Methoxyresorcinol	C_7_H_8_O_3_	140.05	1.63; 1.81	25.12
27	Benzaldehyde	C_7_H_6_O	106.04	1.71; 4.85	0.86
28	DL-Cerulenin	C_12_H_17_NO_3_	223.12	2.65	4.15
29	Methyl isonicotinate	C_7_H_7_NO_2_	137.05	2.84	0.20
30	trans-isoeugenol-O-glucuronide	C_16_H_20_O_8_	340.12	3.03	0.48
31	Acetophenone	C_8_H_8_O	120.06	3.17	0.43
32	Ciclopirox	C_12_H_17_NO_2_	207.13	3.33	0.64
33	2-[4-(3-Hydroxypropyl)−2-methoxyphenoxy]−1,3-propanediol	C_13_H_20_O_5_	256.13	4.14	0.19
34	Eugenol	C_10_H_12_O_2_	164.08	4.14	0.34
35	4-Hydroxybenzaldehyde	C_7_H_6_O_2_	122.04	4.14	1.13
36	4-Propylbenzoic acid	C_10_H_12_O_2_	164.08	4.17	0.29
37	Coumarone	C_8_H_6_O	118.04	4.20	0.21
38	Cianidanol	C_15_H_14_O_6_	290.08	4.35	0.17
39	Viloxazine	C_13_H_19_NO_3_	237.14	4.40	2.14
40	Cuminaldehyde	C_10_H_12_O	148.09	4.51	0.33
41	trans-Anethole	C_10_H_12_O	148.09	4.52	0.37
42	p-cymene	C_10_H_14_	134.11	4.52; 5.14; 6.37	0.50
43	Indane	C_9_H_10_	118.08	4.52; 14.46	0.21
44	(2E)−5-methyl−2-phenylhex−2-enal	C_13_H_16_O	188.12	4.66	0.13
45	p-Isopropyl-α-methylstyrene	C_12_H_16_	160.13	4.75	0.46
46	1,1,6-Trimethyl−1,2-dihydronaphthalene	C_13_H_16_	172.13	4.75	0.18
47	Damascenone	C_13_H_18_O	190.14	4.75; 7.76	0.58
48	2-(2,4-dihydroxyphenyl)−3,5,7-trihydroxy-4H-chromen−4-one	C_15_H_10_O_7_	302.04	5.51	0.38
49	5,7-dihydroxy−2-(2,3,4-trihydroxyphenyl)-4H-chromen−4-one	C_15_H_10_O_7_	302.04	5.69	0.18
50	Methyl cinnamate	C_10_H_10_O_2_	162.07	5.71	0.30
51	4-Vinylcatechol	C_8_H_8_O_2_	136.05	5.88	0.10
52	1-[2-(3-Hydroxy−1-propen−2-yl)−2,3-dihydro−1-benzofuran−5-yl]ethenone	C_13_H_14_O_3_	218.09	5.93; 6.65	0.60
53	Zingerol	C_11_H_16_O_3_	196.11	5.94	1.49
54	Kaempferol	C_15_H_10_O_6_	286.05	5.96	0.25
55	Hymecromone	C_10_H_8_O_3_	176.05	5.96	0.16
56	α-Propylaminopentiophenone	C_14_H_21_NO	219.16	6.01	0.13
57	(3E)−4-(4-Hydroxy−2,6,6-trimethyl−1-cyclohexen−1-yl)−3-buten−2-one	C_13_H_20_O_2_	208.15	6.13	0.17
58	4-[5-(2-Hydroxyethyl)−7-methoxy−3-methyl−2,3-dihydro−1-benzofuran−2-yl]−2-methoxyphenol	C_19_H_22_O_5_	330.15	6.28	0.10
59	β-Ionone	C_13_H_20_O	192.15	6.37	0.28
60	Linalyl propionate	C_13_H_22_O_2_	210.16	6.37	0.27
61	Benzyl formate	C_8_H_8_O_2_	136.05	6.65	0.19
62	10-Deacetylbaccatin III	C_29_H_36_O_10_	544.23	7.70	0.82
63	Favan−3-ol	C_15_H_14_O_2_	226.10	7.97; 9.90; 10.85	0.36
64	1-Hydroxy−1-(4-methoxyphenyl)−3-pentanone	C_12_H_16_O_3_	208.11	8.09	0.12
65	6-Methoxy−3,7-dimethyl−3,4-dihydro-1H-isochromen−8-ol	C_12_H_16_O_3_	208.11	8.09; 10.06	0.79
66	2,2,6,6-Tetramethyl−1-piperidinol (TEMPO)	C_9_H_19_NO	157.15	8.87	0.14
67	3,3′-{[2,4,6-Trihydroxy−5-(2-methylbutanoyl)−1,3-phenylene]bis(methylene)}bis(6-ethyl−4-hydroxy−5-methyl-2H-pyran−2-one)	C_29_H_34_O_10_	542.22	9.18	0.16
68	(7E,13Z)−3-Benzyl−6,12-dihydroxy−4,5,10,12-tetramethyl−1-oxo−2,3,3a,6,6a,9,10,11,12,15-decahydro-1H-cycloundeca[*d*]isoindol−15-yl acetate	C_30_H_39_NO_5_	493.28	9.41	0.18
69	Octinoxate	C_18_H_26_O_3_	290.19	9.84	0.16
70	Benzyl butyl phthalate	C_19_H_20_O_4_	312.14	9.90; 10.85	0.29
71	Phenylamide	C_7_H_7_NO	121.05	10.30	0.20
72	Arborinine	C_16_H_15_NO_4_	285.10	10.30; 10.85; 11.62	1.28
73	(2E)−3-(4-Hydroxy−3-methoxyphenyl)-N-(4-oxobutyl)acrylamide	C_14_H_17_NO_4_	263.12	10.54	0.54
74	(3E)−1-Hydroxy−3-methyl−4-phenyl−3-buten−2-one	C_11_H_12_O_2_	176.08	10.79	0.10
75	Benzamide	C_7_H_7_NO	121.05	10.84; 11.62	0.22
76	N,N-Bis(2-hydroxyethyl)dodecanamide	C_16_H_33_NO_3_	287.25	10.90	0.15
77	1,7-Diphenylheptan−3-one	C_19_H_22_O	266.17	10.93	0.14
78	Norgestrienone	C_20_H_22_O_2_	294.16	10.93	0.26
79	(1aR,2E,4aR,6S,7S,7aR,8 R,11aS)−1,1,3,6-Tetramethyl−9-methylene−4-oxo−1,1a,4,5,6,7,7a,8,9,10,11,11a-dodecahydro−4aH-cyclopenta[*a*]cyclopropa[*f*][11]annulene−4a,7,8-triyl triacetate	C_26_H_36_O_7_	460.25	11.22	0.15
80	Chamazulene	C_14_H_16_	184.13	11.22; 14.46	0.34
81	Safingol	C_18_H_39_NO_2_	301.30	11.37	0.38
82	(8E)−2-Amino−8-octadecene−1,3,4-triol	C_18_H_37_NO_3_	315.28	12.16	0.07
83	12-oxo Phytodienoic Acid	C_18_H_28_O_3_	292.20	12.30	0.30
84	9S,13R−12-Oxophytodienoic acid	C_18_H_28_O_3_	292.20	12.32	0.46
85	α-Eleostearic acid	C_18_H_30_O_2_	278.22	12.43	0.22
86	2-Phenyl−4-pentenal	C_11_H_12_O	160.09	12.51	0.10
87	9-Oxo−10(E),12(*E*)-octadecadienoic acid	C_18_H_30_O_3_	294.22	12.82	0.71
88	Arachidonic acid	C_20_H_32_O_2_	304.24	13.11	0.20
89	2-monolinolenin	C_21_H_36_O_4_	352.26	13.49	0.13
90	(±)-Muscone	C_16_H_30_O	238.23	14.24	0.27
91	L-α-Palmitin	C_19_H_38_O_4_	330.28	14.25	1.64
92	α-Linolenic acid	C_18_H_30_O_2_	278.22	14.30	0.45
93	2-Methylnaphthalene	C_11_H_10_	142.08	14.46	0.15
94	Monoolein	C_21_H_40_O_4_	356.29	14.63	0.10
95	Pheophorbide A	C_35_H_36_N_4_O_5_	592.27	14.71	0.16
96	1-Stearoylglycerol	C_21_H_42_O_4_	358.31	15.27	0.56
97	γ-Linolenic acid ethyl ester	C_20_H_34_O_2_	306.26	15.36	0.10
98	Bis(2-ethylhexyl) amine	C_16_H_35_N	241.28	15.52	0.26
99	Stearamide	C_18_H_37_NO	283.29	15.57; 16.56	0.33
100	N,N-Diethyldodecanamide	C_16_H_33_NO	255.26	15.59	0.16

Building on this overall metabolite distribution, low molecular weight polar compounds were predominantly detected at early retention times and consisted mainly of amino acids and their derivatives, such as valine, proline, pipecolinic acid, pyroglutamic acid, and aminocyclohexanecarboxylic acid. A broad range of aromatic and phenolic compounds was also observed, including benzaldehyde, eugenol, trans-anethole, cuminaldehyde, kaempferol, and several hydroxybenzaldehyde derivatives, which were eluted primarily at mid-retention times. Although most flavonoids and polyphenols were detected at relatively low individual abundances, their consistent presence across the chromatogram highlights the chemical diversity of the extract. More hydrophobic metabolites appeared at later retention times and included fatty acids and glyceride derivatives such as arachidonic acid, α-linolenic acid, γ-linolenic acid ethyl ester, monoolein, 1-stearoylglycerol, and L-α-palmitin. In addition, 10-deacetylbaccatin III, a known taxane precursor, was detected at a retention time of 7.70 min with a relative abundance of 0.82%, confirming the presence of taxane-related compounds in the leaf extract.

While *Taxus* species are well-known sources of paclitaxel (taxol), this specific compound was not detected in our untargeted metabolomic profile. This absence is biologically consistent with the fact that the leaves generally accumulate paclitaxel in mere trace quantities. Previous studies have reported extremely low average taxol yields in the leaves of various *Taxus* species, such as *T. brevifolia* (0.0015%)*, T. baccata* (0.003%)*,* and *T. media* (0.002%) [13]. Such trace concentration often fall below the limit detection (LOD) of an untargeted full-scan UPLC-HRMS approach, which is inherently biased toward highly abundant metabolites. Furthermore, from a physiological perspective, the successful detection of 10-deacetylbaccatin III confirms that the leaves actively synthesize taxane intermediates. It is highly plausible that the leaves primarily serve as a site for generating these essential biosynthesis precursors, while the final biosynthesis and major accumulation of mature paclitaxel occur predominantly in the bark tissues.

### 3.2 Prediction for toxicity

In silico toxicity prediction was performed for 100 metabolites identified in the ethanolic leaf extract of *Taxus sumatrana*, covering mutagenicity, cardiotoxicity, hepatotoxicity, and acute oral toxicity parameters (Table 2). Most compounds were predicted to be non-mutagenic based on the AMES toxicity model, with only a limited subset showing positive AMES alerts, including 4-guanidinobutanal, 4-guanidinobutyric acid, coumarin (2H-chromen−2-one), DL-cerulenin, eugenol, zingerol, and several flavonoid-related structures. Predictions for cardiotoxicity indicated that the vast majority of compounds were non-inhibitors of both hERG I and hERG II channels. Only a small number of metabolites, such as 10-deacetylbaccatin III, γ-linolenic acid ethyl ester, and a limited group of complex aromatic compounds, showed predicted hERG II inhibitory potential, while none were predicted as hERG I inhibitors. Hepatotoxicity prediction further indicated that most metabolites were non-hepatotoxic, with only a few compounds, including hymecromone, arborinine, and selected aromatic amides, flagged for potential liver toxicity.

**Table 2:** Prediction for toxicity profile of compounds identified by UPLCHR-MS/MS.

No.	Compound	AMES toxicity	Max. tolerated dose in human (mg/kg/day)	hERG I inhibitor	hERG II inhibitor	Oral rat acute toxicity LD50 (mg/kgBW)	Hepato-toxicity
1	.alpha.-Aminoadipic acid	No	1.65	No	No	197631	No
2	Choline	No	8.95	No	No	199911	No
3	4-guanidinobutanal	Yes	11.04	No	No	271477	No
4	Dihydrothymine	No	15.45	No	No	275966	No
5	D-(+)-Pipecolinic acid	No	16.98	No	No	218014	No
6	N-Acetyl-S-(allylcarbamothioyl)cysteine	No	25.06	No	No	440760	No
7	DL-Stachydrine	No	11.12	No	No	233388	No
8	Betaine	No	4.46	No	No	201610	No
9	Glycerophosphoglycerol	No	28.12	No	No	415825	No
10	1-Aminocyclohexanecarboxylic acid	No	10.54	No	No	258572	No
11	DL-Carnitine	No	36.22	No	No	224258	No
12	4-Guanidinobutyric acid	Yes	20.42	No	No	209793	No
13	Valine	No	68.23	No	No	166955	No
14	D-(+)-Proline	No	18.66	No	No	192846	No
15	Benzoquinone	No	9.64	No	No	200812	No
16	5-Hydroxymethyl−2-furaldehyde	No	18.49	No	No	296427	No
17	Nicotinic acid	No	14.72	No	No	247049	No
18	Nicotinamide	No	21.18	No	No	287912	No
19	L-Pyroglutamic acid	No	10.52	No	No	233180	No
20	Glycolophenone	No	9.91	No	No	281901	No
21	L-Tyrosine methyl ester	No	6.40	No	No	475822	No
22	2H-chromen−2-one	Yes	1.92	No	No	296455	No
23	m-Anisaldehyde	No	21.09	No	No	249520	No
24	(R)-Salsolinol	No	0.66	No	No	472093	No
25	3,4-Methylenedioxyamphetamine	No	1.92	No	No	441826	Yes
26	2-Methoxyresorcinol	No	11.14	No	No	273232	No
27	Benzaldehyde	No	9.16	No	No	191194	No
28	DL-Cerulenin	Yes	3.99	No	No	492427	No
29	Methyl isonicotinate	No	23.01	No	No	310139	No
30	trans-isoeugenol-O-glucuronide	No	0.48	No	No	830562	No
31	Acetophenone	No	15.42	No	No	207340	No
32	Ciclopirox	No	6.73	No	No	409073	No
33	2-[4-(3-Hydroxypropyl)−2-methoxyphenoxy]−1,3-propanediol	Yes	8.39	No	No	495102	No
34	Eugenol	Yes	10.30	No	No	305688	No
35	4-Hydroxybenzaldehyde	No	8.41	No	No	223693	No
36	4-Propylbenzoic acid	No	3.45	No	No	356554	Yes
37	Coumarone	No	4.79	No	No	268309	No
38	Cianidanol	No	2.40	No	No	628311	No
39	Viloxazine	No	1.34	No	No	595924	No
40	Cuminaldehyde	No	11.04	No	No	238867	No
41	trans-Anethole	No	12.27	No	No	260044	No
42	p-cymene	No	8.00	No	No	245018	No
43	Indane	No	5.07	No	No	188099	No
44	(2E)−5-methyl−2-phenylhex−2-enal	No	3.49	No	No	318299	No
45	p-Isopropyl-α-methylstyrene	No	6.22	No	No	286144	No
46	1,1,6-Trimethyl−1,2-dihydronaphthalene	No	3.85	No	No	298121	No
47	Damascenone	No	4.11	No	No	346237	No
48	2-(2,4-dihydroxyphenyl)−3,5,7-trihydroxy-4H-chromen−4-one	Yes	6.82	No	No	806453	No
49	5,7-dihydroxy−2-(2,3,4-trihydroxyphenyl)-4H-chromen−4-one	Yes	5.12	No	No	779270	No
50	Methyl cinnamate	Yes	5.58	No	No	275030	No
51	4-Vinylcatechol	No	4.73	No	No	282173	No
52	1-[2-(3-Hydroxy−1-propen−2-yl)−2,3-dihydro−1-benzofuran−5-yl]ethanone	No	5.20	No	No	395840	No
53	Zingerol	Yes	8.61	No	No	407124	No
54	Kaempferol	No	8.61	No	No	666205	No
55	Hymecromone	Yes	1.91	No	No	337659	Yes
56	α-Propylaminopentiophenone	No	3.65	No	No	606860	Yes
57	(3E)−4-(4-Hydroxy−2,6,6-trimethyl−1-cyclohexen−1-yl)−3-buten−2-one	No	3.94	No	No	422329	No
58	4-[5-(2-Hydroxyethyl)−7-methoxy−3-methyl−2,3-dihydro−1-benzofuran−2-yl]−2-methoxyphenol	Yes	2.12	No	Yes	734576	No
59	β-Ionone	No	3.58	No	No	333575	No
60	Linalyl propionate	No	3.67	No	No	381023	No
61	Benzyl formate	No	11.61	No	No	268023	No
62	10-Deacetylbaccatin III	No	0.54	No	Yes	2314071	No
63	Favan−3-ol	Yes	1.94	No	No	470060	No
64	1-Hydroxy−1-(4-methoxyphenyl)−3-pentanone	No	8.00	No	No	418717	No
65	6-Methoxy−3,7-dimethyl−3,4-dihydro-1H-isochromen−8-ol	No	8.24	No	No	427041	No
66	2,2,6,6-Tetramethyl−1-piperidinol (TEMPO)	No	7.35	No	No	368509	No
67	3,3′-{[2,4,6-Trihydroxy−5-(2-methylbutanoyl)−1,3-phenylene]bis(methylene)}bis(6-ethyl−4-hydroxy−5-methyl-2H-pyran−2-one)	No	10.26	No	Yes	1399458	No
68	(7E,13Z)−3-Benzyl−6,12-dihydroxy−4,5,10,12-tetramethyl−1-oxo−2,3,3a,6,6a,9,10,11,12,15-decahydro-1H-cycloundeca[*d*]isoindol−15-yl acetate	No	0.03	No	No	1729944	Yes
69	Octinoxate	No	13.09	No	No	494771	No
70	Benzyl butyl phthalate	Yes	11.40	No	No	629266	No
71	Phenylamide	No	16.03	No	No	213658	No
72	Arborinine	Yes	1.57	No	No	589017	Yes
73	(2E)−3-(4-Hydroxy−3-methoxyphenyl)-N-(4-oxobutyl)acrylamide	No	5.81	No	No	554122	No
74	(3E)−1-Hydroxy−3-methyl−4-phenyl−3-buten−2-one	No	11.40	No	No	329981	Yes
75	Benzamide	No	16.03	No	No	213658	No
76	N,N-Bis(2-hydroxyethyl)dodecanamide	No	1.43	No	No	486595	No
77	1,7-Diphenylheptan−3-one	No	7.33	No	No	520357	Yes
78	Norgestrienone	No	0.40	No	No	554789	No
79	(1aR,2E,4aR,6S,7S,7aR,8 R,11aS)−1,1,3,6-Tetramethyl−9-methylene−4-oxo−1,1a,4,5,6,7,7a,8,9,10,11,11a-dodecahydro−4aH-cyclopenta[*a*]cyclopropa[*f*][11]annulene−4a,7,8-triyl triacetate	No	1.10	No	No	1109193	No
80	Chamazulene	No	2.18	No	No	280054	No
81	Safingol	No	0.05	No	No	1034959	No
82	(8E)−2-Amino−8-octadecene−1,3,4-triol	No	0.08	No	No	1222960	No
83	12-oxo Phytodienoic Acid	No	0.06	No	No	736646	No
84	9S,13R−12-Oxophytodienoic acid	No	0.06	No	No	736646	No
85	α-Eleostearic acid	No	0.02	No	No	715872	No
86	2-Phenyl−4-pentenal	No	6.50	No	No	273912	Yes
87	9-Oxo−10(E),12(*E*)-octadecadienoic acid	No	0.06	No	No	751436	No
88	Arachidonic acid	No	0.02	No	No	852785	No
89	2-monolinolenin	No	0.41	No	No	1014160	No
90	(±)-Muscone	No	2.79	No	No	457877	No
91	L-α-Palmitin	No	1.07	No	No	876554	No
92	α-Linolenic acid	No	0.02	No	No	715871	No
93	2-Methylnaphthalene	No	4.22	No	No	277763	No
94	Monoolein	No	0.68	No	No	997974	No
95	Pheophorbide A	No	2.19	No	No	1608008	No
96	1-Stearoylglycerol	No	0.78	No	No	1000037	No
97	γ-Linolenic acid ethyl ester	No	0.51	No	Yes	495521	No
98	Bis(2-ethylhexyl) amine	No	1.34	No	No	540701	No
99	Stearamide	No	0.07	No	No	805386	No
100	N,N-Diethyldodecanamide	No	1.16	No	No	616444	No

Consistent with these findings, predicted maximum tolerated doses (MTD) in humans varied widely among compounds, ranging from very low values below 0.1 mg/kg/day to higher tolerable levels exceeding 60 mg/kg/day. Several endogenous or nutrition-related metabolites, such as amino acids, choline, betaine, carnitine, and organic acids, exhibited relatively high predicted MTD values, indicating favorable safety profiles. Acute oral toxicity predictions in rats showed high LD_50_ values for most compounds, frequently exceeding 200000 mg/kgBW and, in some cases, surpassing 1000000 mg/kgBW, suggesting low acute toxicity potential. Lipid derivatives, fatty acids, glycerides, and sterol-like compounds generally demonstrated particularly high LD₅₀ estimates. Overall, the *in silico* toxicity profiling revealed that the majority of metabolites present in the *T. sumatrana* leaf extract exhibit low predicted acute toxicity and limited safety concerns under the evaluated endpoints.

### 3.3 *In vivo* acute toxicity test

An acute toxicity sighting study was conducted to obtain preliminary safety information and to guide dose selection for the main experiment (Table 3). Across all tested dose levels, ranging from 50 to 2000 mg/kgBW, no mortality was observed within the 24-hour observation period. All animals remained alive and exhibited normal physical appearance and behavior throughout the monitoring intervals at 1, 12, and 24 h post administration. Observations of general activity, feeding behavior, fur condition, fecal output, and bedding moisture did not reveal any abnormal findings in either the treated or placebo groups. These results indicate an absence of acute toxic manifestations following single dose oral administration of the extract across the tested dose range.

**Table 3:** The results of 24-hour observation on the sighting study.

Group	Dose (mg/kgBW)	Number of Mice per Group	Number of Observed Clinical and Behavioral Changes/ Normal for each time point			Number of Death/ Live	Note(s)
			1 h	12 h	24 h		
Placebo	0	1	0/1	0/1	0/1	0/1	No death was found until the highest dose. The main study will be started from the middle dose (300 mg/kgBW).
Dose 1	50	1	0/1	0/1	0/1	0/1	
Dose 2	100	1	0/1	0/1	0/1	0/1	
Dose 3	300	1	0/1	0/1	0/1	0/1	
Dose 4	2000	1	0/1	0/1	0/1	0/1	

The absence of mortality, clinical signs, and behavioural alterations was consistent across all dose groups, including the highest administered dose of 2000 mg/kgBW. No dose dependent trends were detected in any observed parameter during the 24-hour period. Given the complete survival and normal condition of all animals, the extract demonstrated a favourable acute safety profile under the conditions of the sighting study. Based on these outcomes, an intermediate dose of 300 mg/kgBW was selected as the starting dose for the main acute toxicity study. This dose was considered appropriate to balance safety assurance while allowing detection of potential toxic effects at higher exposure levels.

The main acute toxicity study was performed using two dose levels of the *Taxus sumatrana* leaf extract, namely 300 and 2000 mg/kgBW, with a placebo group included as a solvent control (Table 4). Throughout the 24-hour observation period, no mortality was recorded in any experimental group, including those receiving the highest tested dose. All animals remained alive and exhibited normal physical appearance and behavior at each observation point. Clinical assessments conducted at 1, 12, and 24 h post-administration revealed no signs of toxicity, such as changes in locomotor activity, feeding behaviour, posture, fur condition, or excretory patterns. The placebo group similarly showed no abnormal findings, confirming the absence of vehicle-related effects.

**Table 4:** The results of 24-hour observation on the main study.

Group	Dose (mg/kgBW)	Number of Mice per Group	Number of Observed Clinical and Behavioral Changes/Normal for each time point			Number of Death/ Live	Note(s)
			1 h	12 h	24 h		
Placebo	0	5	0/5	0/5	0/5	0/5	No death was found until the highest dose, so that the acute LD_50_> 2000 mg/kgBW.
Dose 3	300	5	0/5	0/5	0/5	0/5	
Dose 4	2000	5	0/5	0/5	0/5	0/5	

Consistent findings across all treated groups indicated that the extract did not induce any observable acute toxic responses within the evaluated timeframe. No dose-related trends were detected in clinical or behavioural parameters between the 300 and 2000 mg/kgBW groups. The complete survival of all animals and the absence of clinical abnormalities support a favorable acute safety profile of the extract following single oral administration. Because no mortality was observed at any dose level, a specific LD_50_ value could not be mathematically calculated via Probit analysis. Based on these observations and following the OECD 420 guidelines, the median lethal dose (LD₅₀) of the *T. sumatrana* leaf extract was estimated to be greater than 2000 mg/kgBW. These results provide a robust basis for subsequent evaluations of blood chemistry and histopathological outcomes in the same experimental model.

### 3.4 The organ-to-body weight rasio

**Fig. 1:**
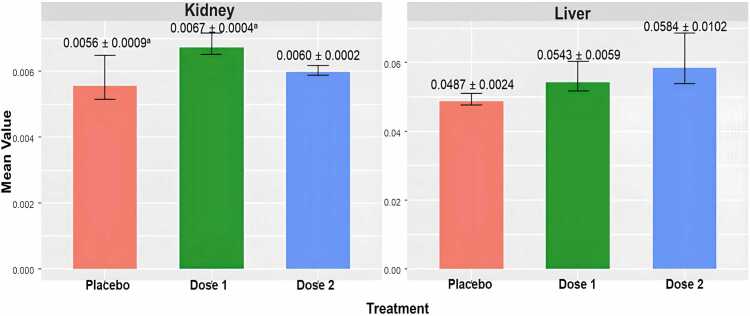
The relative organ-to-body weight ratios of the kidney and liver were calculated for all three groups. The liver relative weight showed an increasing trend across the treatment groups, whereas the kidney relative weight increased in the first dose group and subsequently decreased in the second dose group. A statistically significant difference (P < 0.05) was detected in kidney-to-body weight ratio between the placebo and dose 1 groups, as shown in superscript a.

The relative organ-to-body weight ratios of the liver and kidney are presented in Fig. 1. In the liver, a gradual increase in relative weight was observed across the treatment groups, with mean values rising from the placebo to dose 1 and further to dose 2. Despite this upward trend, statistical analysis did not reveal any significant differences among the groups (p > 0.05). The absence of statistical significance indicates that the observed changes in liver relative weight were within normal physiological variation. These findings suggest that acute exposure to the extract did not induce marked alterations in liver mass relative to body weight within the 24 h observation period.

In contrast, the kidney exhibited a distinct response pattern following treatment. The relative kidney-to-body weight ratio increased significantly in the dose 1 group compared with the placebo group, as indicated by the shared superscript (p < 0.05). However, this increase was not sustained at the higher dose, as the relative kidney weight decreased in the dose 2 group to a level comparable to the placebo. No significant difference was detected between the placebo and dose 2 groups. This non-linear response suggests a transient effect at the intermediate dose rather than a dose-dependent toxic response, which is consistent with the absence of clinical signs and mortality observed in the acute toxicity assessment.

### 3.5 Blood chemistry

**Fig. 2:**
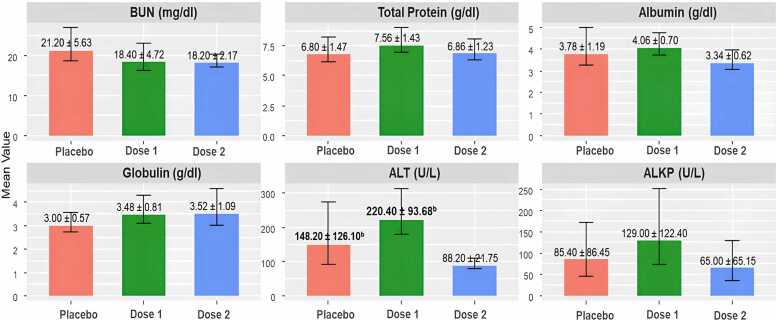
Barplots of selected blood chemistry parameters, presented with standard deviation. Various trends were observed, including constant increases in globulin, decreases in BUN, and peaking and declining in the other parameters. According to statistical analysis, only ALT Dose 1 and Placebo had significant differences (p < 0.05), as shown with superscript b.

The blood chemistry parameters measured after acute exposure are summarized in Fig. 2. Blood urea nitrogen (BUN) levels showed a decreasing trend in the 300 mg/kgBW group compared with the placebo group and remained lower in the 2000 mg/kgBW group. In contrast, globulin concentrations increased progressively with increasing dose levels. Total protein and albumin exhibited a similar pattern, with higher mean values observed in the 300 mg/kgBW group followed by a decline at the 2000 mg/kgBW dose. However, despite these quantitative variations, most parameters remained within comparable ranges across treatment groups.

A comparable dose-related pattern was also observed for liver enzyme parameters. Alanine aminotransferase (ALT) and alkaline phosphatase (ALKP) levels increased at the 300 mg/kgBW dose and subsequently decreased at the highest dose. Statistical analysis identified a significant difference only for ALT between the placebo and the 300 mg/kgBW group (p < 0.05), as indicated by the superscript in Fig. 2. No significant differences were detected for BUN, total protein, albumin, globulin, or ALKP among the treatment groups. Serum creatinine concentrations could not be statistically evaluated because all samples, including those from the placebo group, were below the detectable limit (<0.01 ng/dL), precluding comparative analysis across groups.

### 3.6 Histopathology

Although no deaths occurred in the test animals, histopathological observations showed changes in liver and kidney tissue. Histopathological scoring results showed that the placebo group (0 mg/kgBW) showed no abnormalities in the liver or kidneys, while the 300 mg/kgBW dose only caused mild changes. At a dose of 2000 mg/kgBW, there was a clear increase in various lesions, in the liver in the form of karyolysis, focal necrosis, pyknosis, microvesicular steatosis, hydropic degeneration, and congestion. Meanwhile, in the kidneys, these included karyolysis, focal necrosis, pyknosis, brush border loss, glomerular hypercellularity, and congestion. This confirms the pattern of effects that increase with increasing dose.

**Fig. 3:**
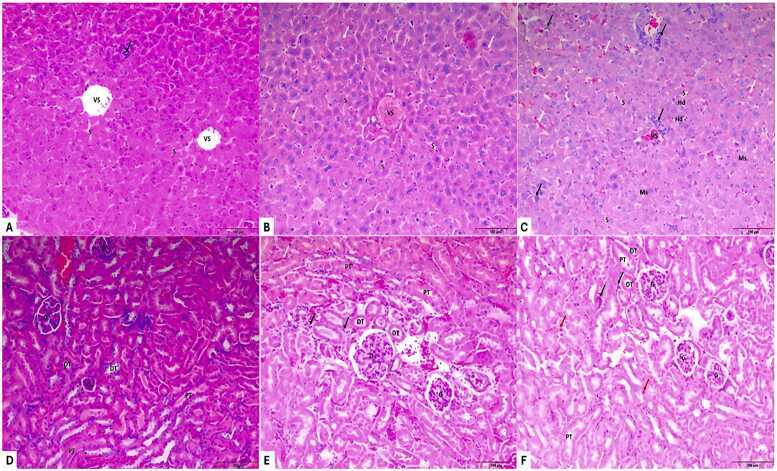
Histopathological features represent each group in the main study. A. Liver Placebo; B. Liver dose 300 mg/kg; C. Liver dose 2000 mg/kg; D. Kidney Placebo; E. Kidney dose 300 mg/kg, F. Kidney dose 2000 mg/kg. VS: vena centralis, S: sinusoid, Hd: hydropic degeneration, Ms: microsteatosis, G: glomerulus, PT: proximal tubule, DT: distal tubule, back arrow: polymorphonuclear cell infiltration, white arrow: erythrocyte, red arrow: pycnotic nucleus.

**Fig. 4:**
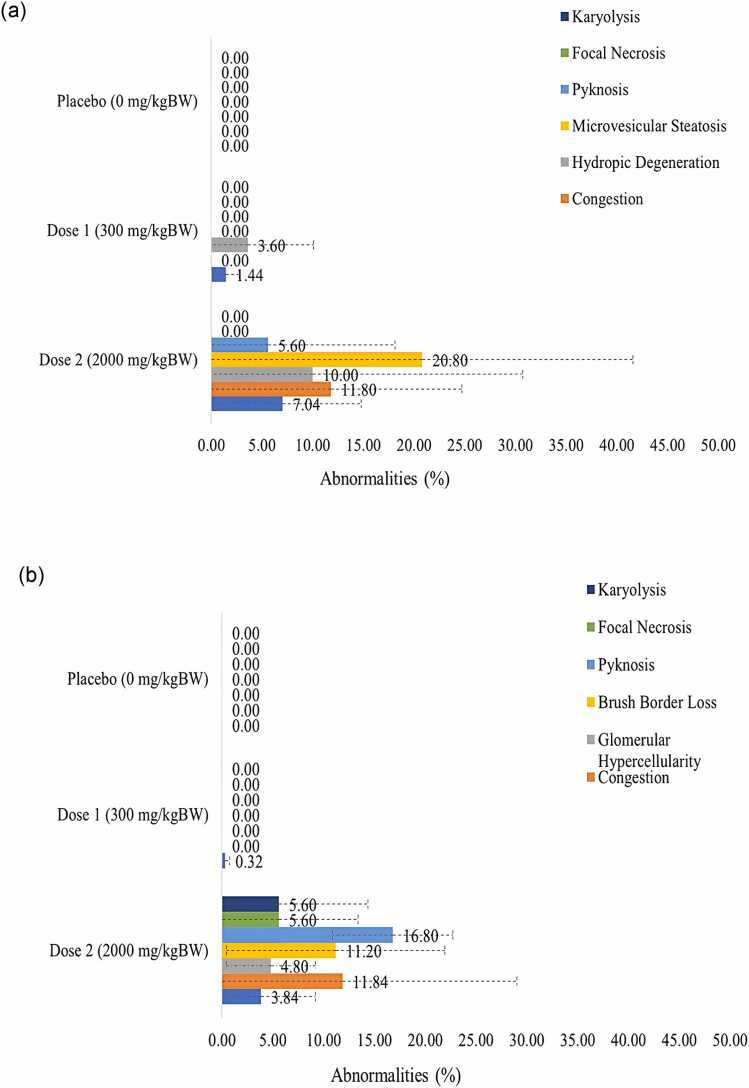
Histopathological scoring for abnormalities of (A) liver and (B) kidney in the main study.

Based on the histopathology scoring (Fig. 4), the placebo group showed an unaffected liver and kidney picture, characterized by the absence of indicators of cell nuclear damage or degenerative and vascular changes. At low doses, histological changes were still very minimal and limited, mainly consisting of mild findings that indicate initial cellular stress (cell nuclear changes), without being followed by prominent cytoplasmic degeneration or circulatory disorders. At high doses, a clear and more extensive increase in various parameters of tissue damage was observed. In the liver, the lesion pattern reflected a combination of parenchymal cell injury and metabolic disorders (cell nuclear changes consistent with cell death, the presence of focal necrosis, microvesicular steatosis, and hydropic degeneration) accompanied by a hemodynamic component in the form of congestion, thus overall depicting hepatotoxicity with a degenerative-necrotic spectrum and contributing to blood flow disorders. In the kidney, prominent lesions at high doses indicate primary involvement of the tubular and glomerular compartments, with nuclear changes and focal necrosis indicating cell injury, along with brush border loss indicating proximal tubular epithelial damage, and glomerular hypercellularity and congestion reflecting an inflammatory/reactive response and microcirculatory disturbance; overall, these findings are consistent with dose-dependent nephrotoxicity affecting both the filtration and reabsorption units.

## 4 Discussion

Metabolomic profiling using UPLC-HRMS/MS revealed that *T. sumatrana* leaf extract contains a wide range of bioactive compounds, with a total of 100 metabolites identified. Among these, 2-methoxyresorcinol, choline, and coumarin (2H-chromen−2-one) were the most abundant, showing relative proportions of 25.12%, 9.21%, and 8.33%, respectively. 2-Methoxyresorcinol, a naturally occurring plant metabolite, has been reported to possess antioxidant properties and tumor growth inhibitory effects. Structurally related compounds, such as 4-methoxyresorcinol, have also demonstrated anticancer potential. For example, 4-methoxyresorcinol isolated from *Melia azedarach* L. leaves exhibited moderate cytotoxicity against LNCap, MDA-MB−231, and MCF7 cancer cell lines [22]. The antioxidant activity of 2-methoxyresorcinol likely reduces oxidative stress caused by other metabolites, protecting organs from damage. Long-term studies in rats indicated that 2-methoxyresorcinol caused only weight loss without microscopic liver or kidney damage over 180 days [23]. Similarly, phenolic derivatives such as 5-methylresorcinol act as free radical scavengers, neutralizing reactive oxygen species and contributing to mild and non-progressive toxicological effects [24]. These observations suggest that 2-methoxyresorcinol plays a key role in both the therapeutic potential and relative safety of *T. sumatrana* leaf extract.

Choline, the second major metabolite, is a vitamin-like nutrient and a precursor of acetylcholine, membrane phospholipids, and betaine. It supports critical functions in the liver, brain, muscle, and kidney. Studies have shown that higher dietary choline intake is associated with improved cognitive performance and slower cognitive decline in middle-aged and older adults. In animal models, chronic choline supplementation improves memory and neurochemical status in the brain [25], [26]. Given these biological roles, choline present in *T. sumatrana* leaf extract may contribute to neuroprotective support and everall neurological health, and warrants further investigation in disease-specific experimental models.

Coumarin, present at 8.33%, adds further pharmacological benefits, including anticancer and immunomodulatory effects. Previous studies reported that extracts from bark, leaf, and shoots of *T. sumatrana* exhibited cytotoxicity against HELA, T47D, and MCF−7/HER2 cells [12]. In this study, histopathology showed pyknosis in kidney tissue, an early sign of apoptosis, which may result from mitochondrial damage, oxidative stress, or glutathione depletion caused by coumarin metabolites such as coumarin 3,4-epoxide [27]. Apoptosis induction is a key mechanism in cancer therapy, restoring controlled cell death in tumor cells. Although coumarin can exert anticoagulant effects at high doses, its low concentration in the extract likely explains the minimal congestion observed in liver and kidney tissues. Therefore, the observed nuclear changes may reflect a localized cellular response rather than widespread tissue injury.

Metabolomic analysis also detected 10-deacetylbaccatin III at 0.82% abundance. Baccatine-type compounds are well known for their anticancer potential and act as intermediates in the biosynthesis of clinically used taxanes, such as paclitaxel (Taxol®) and docetaxel (Taxotere®). Taxane diterpenoids structurally related to baccatin have shown cytotoxic effects against breast, ovarian, and lung cancer cell lines [28]. Although present in low abundance, these compounds likely contribute to the extract’s overall pharmacological activity, supporting its therapeutic potential.

Toxicity assessment using in silico predictions confirmed that the major metabolites are safe at the doses tested (Table 2). In the in vivo acute toxicity study, mice receiving a single oral dose of 2000 mg/kgBW (the highest dose) showed no signs of toxicity, aligning with computational predictions where all compounds had toxicity thresholds above the administered dose. The hERG I channel inhibition analysis indicated no interference with cardiac potassium ion flow, and although four compounds showed potential hERG II inhibition, their oral rat LD_50_ values exceeded 2000 mg/kgBW. These results indicate that *T. sumatrana* leaf extract has low acute toxicity and does not pose immediate cardiac risks. However, this evaluation was intentionally focused on 24 h window to capture immediate organ stress and metabolomic shifts exactly before the inset of natural physiological recovery, which could otherwise mask early tissue alterations. To complement these findings and fully rule out delayed toxic manifestation, future studies utilizing a conventional 14 days observation protocol remain highly recommended to evaluate long-term recovery mechanism.

Blood chemistry and histopathology analyses indicate that *T. sumatrana* extract induces a dose-dependent biological response, with a clear distinction between the extent of systemic biochemical changes and tissue structural changes. Blood chemistry results, such as BUN, total protein, albumin, and ALP, did not show consistent significant changes at any dose (Fig. 2). This indicates that the liver and kidneys are still functioning correctly. A significant increase in ALT levels at low doses, but not sustained at higher doses, suggests a transient hepatic response due to metabolic adaptation or enzyme induction, rather than progressive hepatotoxicity with a linear dose-response pattern. This biphasic, non linear progression is also reflected in the relative kidney weights, pointing toward a shared mechanism of hormesis and adaptive celluler responses across these target organs. The intermediate exposure at 300 mg/kgBW appears to represent a threshold of initial metabolic challenge, triggering transient cellular stress. Conversely, the maximum dose of 2000 mg/kgBW introduces a significantly higher concentration of bioactive constituent that likely upregulate endogenus cytoprptective pathways. This interpretation is strongly supported by our metabolomic profiling, which reveals that *T. sumaterana* leaf extract is exceptionally rich in potent antioxidant compounds, such as 2-methoxyresorcinol. At high doses, the dense availability of these antioxidants effectively counteracts oxidative stress, nutralizing further enzyme leakage and stabilizing tissue weight, which demonstrates a robust physiological adaptation rather than escalating damage [29].

Histopathology results showed clear changes in liver and kidney tissue at high doses. In the liver, microvesicular steatosis, hydropic degeneration, and sinusoidal congestion were found, indicating impaired lipid metabolism and intracellular ion balance. Meanwhile, histological changes in the kidneys were characterized by loss of the brush border in the proximal tubules, pyknosis, congestion, and focal necrosis, indicating tubular injury. The loss of the brush border is a sensitive indicator of impaired proximal tubular reabsorption function and often appears earlier than changes in renal biomarkers in the blood. The absence of an increase in BUN in this study indicates that the kidneys are still functioning well. Nuclear changes, such as pyknosis and karyolysis, observed in the liver and kidneys, indicate that some hepatocytes have experienced severe injury, potentially leading to cell death through apoptosis or necrosis, especially with high doses of *T. sumatrana* extract. These effects are likely multifactorial and reflect the combined action of bioactive metabolites rather than a single compound.

Despite these observed structural changes, it is crucial to emphasize that the maximum histopathological abnormality recorded at the extreme limit dose of 2000 mg/kgBB was anly approximately 20%. These alterations primarily consist of mild and potentially reversible tissue injury, indicating a high threshold of physiological tolerance. In stark contrast, existing toxicological data on the European yew (*T. baccata*) reveals extreme acute toxicity, particularly following the ingestion of its leaves (needles). The lethal dose for an adult is reported to be 50 g of yew needles [30], [31]. Estimating that 1 g of yew needles contains approximately 5 mg of taxines, the minimal toxic dose for humans is calculated to be 3.0–6.5 mg taxines/kg [30]. Ingestion of these needles frequently leads to life-threatening cardiotoxicity, such as refractory ventricular fibrillation and asystole [30]. The complete absence of lethal systemic toxicity, severe organ failure, or cardiac arrest in our study at a massive dose of 2000 mg/kgBW demonstrates that *T. sumatrana* leaves are inherently far less toxic than *T. baccata* leaves. This robust safety profile further supports the promising and safe potential of *T. sumatrana* leaf extract for future pharmacological applications.

Despite these structural changes, ALT and ALKP levels, conventional indicators of liver injury [32] remained within physiological ranges, suggesting preserved liver function. The hepatoprotective and antioxidant effects of choline and 2-methoxyresorcinol likely mitigated oxidative stress, while the limited abundance of coumarin minimized apoptotic and anticoagulant effects. Minor constituents such as 10-deacetylbaccatin III, despite their cytotoxic potential in vitro, did not induce observable histological or biochemical alterations at the tested doses. Overall blood chemistry profiles remained stable, supporting the conclusion that acute exposure did not compromise essential organ function.

Overall, these findings demonstrate that *T. sumatrana* leaf extract exhibits a low acute toxicity with mild tissue alterationsprofile in vivo. The combination of antioxidant, neuroprotective, and anticancer metabolites ensures both pharmacological benefit and minimal toxicity. Even at high acute doses, the extract induced only limited and non-lethal structural changes in liver and kidney tissues, highlighting its promise for further pharmacological development and safety evaluation.

While the current study established a robust acute safety profile for *T. sumatrana* leaves extract, it is important to acknowledge certain limitations regarding the pharmacokinetics of its bioactive constituents. This study focused strictly on toxicological endpoints. Consequently, the basic ADME (absorption, distribution, metabolism, and excretion) profiles and the systemic bioavailability of the identified metabolites were not evaluated. The in silico assessments conducted herein were specifically utilized as a validation layer for toxicity prediction, linking the metabolomic profile directly to the in vivo safety findings rather than determining pharmacokinetic efficacy. Fully elucidating the ADME profiles and the bioavailability of these precursors remains a critical next stop for future pharmacological studies.

## 5 Conclusion

The orally administered ethanolic leaf extract of *T. sumatrana* is low acute toxicity with mild tissue alterations in acute exposure, with an LD₅₀ exceeding 2000 mg/kg BW and minimal effects on organ-to- body weight ratios, blood chemistry, and histopathology, indicating good tolerability. Metabolomic profiling using UPLC-HRMS/MS successfully identified 100 compounds, and in silico toxicity predictions demonstrated that the majority of these compounds are non-AMES toxic, non-inhibitors of hERG I and II, and possess predicted acute LD₅₀ values above 2000 mg/kg BW. Together, these findings provide strong evidence that the extract has a low acute toxicity risk. However, while acute toxicity is absent, prolonged or repeated administration may still affect organ function. Since acute safety does not always guarantee safety in long-long term therapy (i.e. cancer therapy), this highlights the need for sub-acute (28 days), sub-chronic (90 days), and chronic toxicity (minimum 6 months) studies to establish a comprehensive safety profile. These results support the potential use of *T. sumatrana* leaves as a sustainable alternative to bark harvesting for pharmacological applications, particularly as a source of bioactive taxane derivatives, without compromising tree survival or safety in acute use.

## Declaration of Competing Interest

The authors declare that they have no known competing financial interests or personal relationships that could have appeared to influence the work reported in this paper.

## Data Availability

Data will be made available on request.
