# Supplementary material for: Acute toxicity assessment and metabolomic profiling of Taxus sumatrana leaf extract as a sustainable alternative to bark harvesting
Source: Toxicol Rep. 2026 Jun 6;16:102289. doi: 10.1016/j.toxrep.2026.102289 (PMC13273224; doi:10.1016/j.toxrep.2026.102289)
Supplement: Supplementary file 1 — Supplementary material [file mmc1.pdf]

KEMENTERIAN LINGKUNGAN HIDUP DAN KEHUTANAN
DIREKTORAT JENDERAL KONSERVASI SUMBER DAYA ALAM DAN EKOSISTEM

KEPUTUSAN DIREKTUR JENDERAL KONSERVASI SUMBER DAYA ALAM DAN EKOSISTEM
NOMOR : SK.144/KSDAE/SETKSDAE/KSA.2/7/2024

TENTANG

IZIN AKSES SUMBER DAYA GENETIK SPESIES LIAR UNTUK KEPENTINGAN PENELITIAN KEPADA BOGOR NATUR INDONESIA ATAS NAMA DR. WANDA KUSWANDA, S.HUT., M.SC DAN TIM

DIREKTUR JENDERAL KONSERVASI SUMBER DAYA ALAM DAN EKOSISTEM,

Menimbang :

a. bahwa dengan surat No: 062/BNGi-Bioprospeksi/VII/2024 tanggal 12 Juli 2024, Ketua Bogor Natur Indonesia (BNGi) pada intinya menyampaikan permohonan izin akses sumber daya genetik jenis dilindungi untuk kepentingan penelitian atas nama Dr. Wanda Kuswanda, S.Hut., M.Sc dan Tim dengan judul "Pengembangan Bioprospeksi dari Kawasan Konservasi Taman Nasional Kerinci Seblat (Sub Kegiatan: Eksplorasi Taksus)".

b. bahwa setelah dilakukan kajian teknis dan telaahan hukum, permohonan Dr. Wanda Kuswanda, S.Hut., M.Sc dan Tim dari Bogor Natur Indonesia, dinilai dapat dipertimbangkan untuk diberikan Izin Akses Sumber Daya Genetik Spesies Liar untuk kepentingan penelitian sebagaimana dimaksud pada huruf a;

c. bahwa berdasarkan pertimbangan sebagaimana dimaksud pada huruf a dan huruf b, maka perlu menetapkan Keputusan Direktur Jenderal Konservasi Sumber Daya Alam dan Ekosistem tentang Izin Akses Sumber Daya Genetik untuk Kepentingan Penelitian kepada Bogor Natur Indonesia Atas Nama Dr. Wanda Kuswanda, S.Hut., M.Sc dan Tim.

Mengingat :

1. Undang-undang Nomor 5 Tahun 1990 tentang Konservasi Sumber Daya Alam Hayati dan Ekosistemnya;
2. Undang-undang Nomor 5 Tahun 1994 tentang Pengesahan Konvensi PBB mengenai Keanekaragaman Hayati;
3. Undang-undang Nomor 41 Tahun 1999 tentang Kehutanan yang telah diubah dengan Undang-undang Nomor 19 Tahun 2004 tentang Penetapan Peraturan Pemerintah Pengganti Undang-undang Nomor 1 Tahun 2004 Tentang Perubahan Atas Undang-undang Nomor 41 Tahun 1999 tentang Kehutanan Menjadi Undang-undang;
4. Undang-undang Nomor 32 Tahun 2009 tentang Perlindungan dan Pengelolaan Lingkungan Hidup;

5. Undang-undang....

5. Undang-undang Nomor 11 Tahun 2013 tentang Pengesahan *Nagoya Protocol on Access to Genetic Resources and the Fair and Equitable Sharing of Benefit Arising from their Utilization to the Convention on Biological Diversity;*
6. Peraturan Pemerintah Nomor 7 Tahun 1999 tentang Pengawetan Jenis Tumbuhan dan Satwa;
7. Peraturan Pemerintah Nomor 8 Tahun 1999 tentang Pemanfaatan Jenis Tumbuhan dan Satwa Liar;
8. Peraturan Pemerintah RI Nomor 41 Tahun 2006 tentang Perizinan Melakukan Kegiatan Penelitian dan Pengembangan bagi Perguruan Tinggi Asing, Lembaga Penelitian dan Pengembangan Asing, Badan Usaha Asing dan Orang Asing;
9. Peraturan Pemerintah Nomor 12 Tahun 2014 tentang Jenis dan Tarif Atas Jenis Penerimaan Negara Bukan Pajak yang Berlaku pada Kementerian Kehutanan;
10. Peraturan Presiden Nomor 16 Tahun 2015 tentang Kementerian Lingkungan Hidup dan Kehutanan;
11. Peraturan Menteri Lingkungan Hidup dan Kehutanan Nomor P.2/MenLHK/Setjen/Kum.1/1/2018 tentang Akses Pada Sumber daya Genetik Spesies Liar Dan Pembagian Keuntungan Atas Pemanfaatannya;
12. Peraturan Menteri Lingkungan Hidup dan Kehutanan Nomor P.106/MenLHK/Setjen/Kum.1/12/2018 tentang Perubahan Kedua Atas Peraturan Menteri Lingkungan Hidup dan Kehutanan Nomor P.20/MenLHK/Setjen/Kum.1/6/2018 tentang Jenis Tumbuhan dan Satwa yang Dilindungi;
13. Peraturan Menteri Lingkungan Hidup dan Kehutanan Nomor P.15 Tahun 2021 tentang Organisasi dan Tata Kerja Kementerian Lingkungan Hidup dan Kehutanan.

Memperhatikan : a. Nota Dinas Direktur Konservasi Keanekaragaman Hayati Spesies dan Genetik Nomor: ND.750/KKHSG/PSG1/KSA.2.2/B/07/2024 tanggal 16 Juli 2024

b. Surat Rekomendasi Direktur Sekretariat Kewenangan Ilmiah Keanekaragaman Hayati - BRIN melalui surat Nomor B-6089/IV/KS.00/7/2024 tanggal 12 Juli 2024.

M E M U T U S K A N :

Menetapkan : KEPUTUSAN DIREKTUR JENDERAL KONSERVASI SUMBER DAYA ALAM DAN EKOSISTEM TENTANG IZIN AKSES SUMBER DAYA GENETIK UNTUK KEPENTINGAN PENELITIAN KEPADA BOGOR NATUR INDONESIA ATAS NAMA Dr. WANDA KUSWANDA, S.Hut., M.Sc

KESATU....

KESATU : Memberikan Izin Akses Sumber Daya Genetik Spesies Liar kepada Bogor Natur Indonesia yang diketuai oleh Dr. Wanda Kuswanda, S.Hut., M.Sc dengan anggota sebagai berikut:

1. Muhammad Iman Surya, Ph.D. – BRIN
2. Hendra Helmanto, S.Hut., M.Sc. – BRIN
3. M. Hadi Saputra, S.Hut.,M.P.W.K.,M.Sc. – BRIN
4. Pamungkas Rizki Ferdian, M.Si. – BRIN
5. Dedi, S.Hut. – TN Kerinci Seblat
6. Eko Supriyatno – TN Kerinci Seblat

KEDUA : Izin Akses Sumber Daya Genetik Spesies Liar sebagaimana dimaksud Amar KESATU meliputi kegiatan untuk memperoleh dan atau mengambil, membawa dan memanfaatkan sumber daya genetik dengan jenis, bentuk, jumlah dan lokasi pengambilan sebagai berikut:

Nama Jenis	Bentuk Spesimen	Jumlah Spesimen	Lokasi Pengambilan
Taksus (*Taxus sumatrana*)	Daun	10 kg basah	Taman Nasional Kerinci Seblat
	Kulit	10 kg basah	
	Ranting	10 kg basah	

KETIGA : Spesimen tumbuhan taksus (*Taxus sumatrana*) sebagaimana AMAR KEDUA hanya dapat dibawa/diangkut dari lokasi pengambilan ke Laboratorium Genomik dan Laboratorium Cara Pembuatan Obat Tradisional yang Baik (CPTB) BRIN - Cibinong untuk dilakukan analisis dengan metode yang sesuai dengan kaidah ilmiah.

KEEMPAT : Pemegang izin wajib memenuhi ketentuan sebagai berikut:

a. Pelaksanaan izin akses sumber daya genetik sebagaimana dimaksud Amar KESATU agar dikoordinasikan dengan Balai Konservasi Sumber Daya Alam setempat, serta dilakukan dengan menggunakan alat dan cara angkut yang layak (aman) sesuai dengan ketentuan peraturan perundang-undangan.
b. Setiap pengangkutan sampel dilengkapi SATS-DN (Surat Izin Angkut Tumbuhan dan Satwa Liar Dalam Negeri) yang diterbitkan oleh Balai Konservasi Sumber Daya Alam setempat.
c. Untuk menghindari kemungkinan replikasi *Deoxyribose-nucleic acid* (DNA), setelah masa penelitian berakhir maka material sisa sampel harus dihancurkan dengan membuat berita acara pelaporan pemusnahan sampel atau menyimpan sisa material di lokasi yang disetujui oleh Direktur Jenderal Konservasi Sumber Daya Alam dan Ekosistem.
d. Tidak memindahtangankan dan atau memperjualbelikan sampel tersebut kepada pihak lain.
e. Tidak memindahtangankan izin mengambil dan mengedarkan sampel ini kepada pihak lain.
f. Segala bentuk komersialisasi dari pemanfaatan sampel penelitian ini tidak dibenarkan.

g. Menyampaikan ...

g. Menyampaikan hasil penelitian kepada Direktur Jenderal Konservasi Sumber Daya Alam dan Ekosistem, Direktur Konservasi Keanekaragaman Hayati Spesies dan Genetik dan Kepala Unit Pelaksana Teknis sebagaimana pada huruf a, paling lama 1 (satu) bulan setelah selesainya pelaksanaan kegiatan penelitian.

h. Membayar pungutan yang ditetapkan sesuai ketentuan perundang-undangan.

i. Tidak diperbolehkan membawa/mengangkut sampel ke lokasi lain selain lokasi yang telah tercantum sebagaimana dimaksud Amar KETIGA.

KELIMA	:	Izin akses sumber daya genetik satwa liar sebagaimana dimaksud Amar KESATU, berlaku 12 (dua belas) bulan sejak tanggal ditetapkan dan hanya dipergunakan untuk 1 (satu) kali penelitian dan dapat diperpanjang berdasarkan permohonan dengan ketentuan permohonan perpanjangan disampaikan kepada Direktur Jenderal Konservasi Sumber Daya Alam dan Ekosistem paling lambat 6 (enam) bulan sebelum masa berlaku izin ini berakhir.
KEENAM	:	Direktur Konservasi Keanekaragaman Hayati Spesies dan Genetik berkewajiban melakukan monitoring dan evaluasi atas pelaksanaan keputusan ini.
KETUJUH	:	Apabila pemegang izin tidak memenuhi ketentuan sebagaimana dimaksud dalam keputusan ini, maka akan dikenakan sanksi sesuai dengan ketentuan peraturan perundang-undangan.
KEDELAPAN	:	Keputusan ini mulai berlaku sejak tanggal ditetapkan.

Ditetapkan di : Jakarta
pada tanggal : 23 JULI 2024

DIREKTUR JENDERAL,

Prof. Dr. Satyawan Pudyatmoko, S. Hut., M.Sc
NIP. 19710809 199512 1 001

Salinan keputusan ini disampaikan kepada:
1. Sekretaris Jenderal Kementerian Lingkungan Hidup dan Kehutanan;
2. Inspektur Jenderal Kementerian Lingkungan Hidup dan Kehutanan;
3. Sekretaris Direktorat Jenderal Konservasi Sumber Daya Alam dan Ekosistem;
4. Direktur Konservasi Keanekaragaman Hayati Spesies dan Genetik;
5. Direktur Sekretariat Kewenangan Ilmiah Keanekaragaman Hayati – BRIN;
6. Kepala Balai Besar Taman Nasional Kerinci Seblat;
7. Kepala Balai Konservasi Sumber Daya Alam Jambi;
8. Kepala Balai Konservasi Sumber Daya Alam Sumatera Barat;
9. Kepala Balai Konservasi Sumber Daya Alam Sumatera Selatan;
10. Kepala Balai Konservasi Sumber Daya Alam Bengkulu;
11. Yang Bersangkutan.
