# Supplementary material for: Acute toxicity assessment and metabolomic profiling of Taxus sumatrana leaf extract as a sustainable alternative to bark harvesting
Source: Toxicol Rep. 2026 Jun 6;16:102289. doi: 10.1016/j.toxrep.2026.102289 (PMC13273224; doi:10.1016/j.toxrep.2026.102289)
Supplement: Supplementary file 2 — Supplementary material [file mmc2.pdf]

KOMISI ETIK BIDANG PEMELIHARAAN DAN PENGGUNAAN HEWAN
BADAN RISET DAN INOVASI NASIONAL
Gedung B.J. Habibie Lantai 8 Jalan M.H. Thamrin No. 8, Jakarta Pusat 10340
Laman: https://klirensetik.brin.go.id/, klirensetik@brin.go.id

SURAT KEPUTUSAN KLIRENS ETIK
Riset Bidang Pemeliharaan dan Penggunaan Hewan
Nomor: 097/KE.02/SK/05/2025

Komisi Etik Bidang Pemeliharaan dan Penggunaan Hewan BRIN menerangkan bahwa,

Judul Riset	:	Uji Praklinis Ekstrak Daun *Taxus sumatrana*: Toksisitas Akut, Efikasi Anti-Diabetes, Iritasi Kulit, dan Toksisitas Sub-Kronis
Nomor Usulan	:	22042025000007
Unit/Lembaga	:	Badan Riset dan Inovasi Nasional (BRIN)
Koordinator Periset	:	Wanda Kuswanda

Telah disidangkan pada tanggal 9 Mei 2025

Berdasarkan hasil sidang tersebut dan revisi yang telah dilakukan sesuai rekomendasi sidang, Komisi Etik Bidang Pemeliharaan dan Penggunaan Hewan BRIN memutuskan: **Riset dengan nomor usulan di atas telah memenuhi persyaratan Klirens Etik dengan jangka waktu riset mulai Juni 2025 sampai dengan Mei 2026**

Periset tetap berkewajiban untuk:
a. Mengajukan permohonan baru apabila ada amandemen rancangan atau subyek riset;
b. Memberikan laporan apabila riset lapangan telah selesai;
c. Mengajukan amandemen bila ada perubahan lokasi, waktu riset dan/atau dihentikan sebelum waktunya.

Komisi Etik Bidang Pemeliharaan dan Penggunaan Hewan BRIN mempunyai hak untuk melakukan pemantauan selama riset berlangsung.

Jakarta, 28 Mei 2025
Ketua Komisi Etik
Bidang Pemeliharaan dan Penggunaan
Hewan BRIN,

TT ELEKTRONIK

Prof. Dr. Andria Agusta

Dokumen ini ditandatangani secara elektronik menggunakan sertifikat dari BSrE, silahkan lakukan verifikasi pada dokumen elektronik yang dapat diunduh dengan melakukan scan QR Code
